# Balance of Na^+^, K^+^, and Cl^–^ Unidirectional Fluxes in Normal and Apoptotic U937 Cells Computed With All Main Types of Cotransporters

**DOI:** 10.3389/fcell.2020.591872

**Published:** 2020-11-06

**Authors:** Valentina E. Yurinskaya, Igor A. Vereninov, Alexey A. Vereninov

**Affiliations:** ^1^Laboratory of Cell Physiology, Institute of Cytology, Russian Academy of Sciences, St-Petersburg, Russia; ^2^Peter the Great St-Petersburg Polytechnic University, St-Petersburg, Russia

**Keywords:** cell ion homeostasis, membrane transport, ion channels, sodium pump, cotransporters, ion fluxes calculation, apoptosis

## Abstract

Fluxes of monovalent ions through the multiple pathways of the plasma membrane are highly interdependent, and their assessment by direct measurement is difficult or even impossible. Computation of the entire flux balance helps to identify partial flows and study the functional expression of individual transporters. Our previous computation of unidirectional fluxes in real cells ignored the ubiquitous cotransporters NKCC and KCC. Here, we present an analysis of the entire balance of unidirectional Na^+^, K^+^, and Cl^–^ fluxes through the plasma membrane in human lymphoid U937 cells, taking into account not only the Na/K pump and electroconductive channels but all major types of cotransporters NC, NKCC, and KCC. Our calculations use flux equations based on the fundamental principles of macroscopic electroneutrality of the system, water balance, and the generally accepted thermodynamic dependence of ion fluxes on the driving force, and they do not depend on hypotheses about the molecular structure of the channel and transporters. A complete list of the major inward and outward Na^+^, K^+^, and Cl^–^ fluxes is obtained for human lymphoid U937 cells at rest and during changes in the ion and water balance for the first 4 h of staurosporine-induced apoptosis. It is shown how the problem of the inevitable multiplicity of solutions to the flux equations, which arises with an increase in the number of ion pathways, can be solved in real cases by analyzing the ratio of ouabain-sensitive and ouabain-resistant parts of K^+^ (Rb^+^) influx (OSOR) and using additional experimental data on the effects of specific inhibitors. It is found that dynamics of changes in the membrane channels and transporters underlying apoptotic changes in the content of ions and water in cells, calculated without taking into account the KCC and NKCC cotransporters, differs only in details from that calculated for cells with KCC and NKCC. The developed approach to the assessment of unidirectional fluxes may be useful for understanding functional expression of ion channels and transporters in other cells under various conditions. Attached software allows reproduction of all calculated data under presented conditions and to study the effects of the condition variation.

## Introduction

Apoptosis is one of the three main types of cell death, along with autophagy and necrosis itself (Galluzzi et al., 2018). A hallmark of apoptosis is a specific cell shrinkage or, at least, the absence of swelling and rupture of the plasma membrane (Yurinskaya et al., 2005). This is due to specific apoptotic changes in monovalent ion homeostasis, which is closely related to cell water balance regulation (Maeno et al., 2000, 2012; Okada et al., 2001; Lang et al., 2005; Lang and Hoffmann, 2012, 2013). Until now, the quantitative study of changes in channels and transporters responsible for specific apoptotic changes in the balance of Na^+^, K^+^, and Cl^–^ has been based on the use of staurosporine-treated U937 cells and computer modeling without considering KCC and NKCC cotransporters (Yurinskaya et al., 2019). However, these cation-coupled Cl^–^ cotransporters of the gene family SLC12 attract much attention in the recent decade (Gagnon and Delpire, 2013; Jentsch, 2016; Delpire and Gagnon, 2018). They can transport Cl^–^ against an electrochemical gradient and create a non-equilibrium distribution of Cl^–^ across the membrane. This is what makes Cl^–^ an active player in various physiological processes (Jentsch, 2016) since the permeability of the Cl^–^ channels can affect the entire homeostasis of monovalent ions only when Cl^–^ is in a non-equilibrium state. The interplay of various Cl^–^ coupled cotransporters and Cl^–^ channels has been particularly intensively studied in connection with signal transmission in neurons (Kaila et al., 2014; Doyon et al., 2016; Currin et al., 2020; Wilke et al., 2020). Progress in the molecular biology of the cation-coupled Cl^–^ cotransporters is exciting; however, their functional expression and role in maintaining Cl^–^ homeostasis in non-polarized cells is investigated much worse because electrophysiological methods cannot be applied here, and possible tools are rather limited. It was said: “Electrical activity in neurons requires a seamless functional coupling between plasmalemmal ion channels and ion transporters. Although ion channels have been studied intensively for several decades, research on ion transporters is in its infancy” (Kaila et al., 2014).

In our previous study, the cells with the sodium pump, electroconductive Na^+^, K^+^, and Cl^–^ channels and only one cotransporter NC were considered (Yurinskaya et al., 2019). It was found that the permeability of Cl^–^ channels significantly changes at the early stage of apoptosis. How the presence of KCC and NKCC will affect the behavior of cells and how the parameters of the main ion pathways obtained by calculations will change were unknown. An increase in the number of considered ionic paths significantly increases the difficulties of finding the parameters, and these problems are also the subject of the current study. We believe that the developed approach is a useful tool for studying the fluxes of monovalent ions across the plasma membrane with all major ion pathways.

## Materials and Methods

### Cell Cultures

U937 human histiocytic lymphoma cells were obtained from the Russian Cell Culture Collection (Institute of Cytology, Russian Academy of Sciences, cat. number 160B2). The cells were cultured in RPMI 1640 medium supplemented with 10% fetal bovine serum (FBS) at 37°C and 5% CO_2_. For the induction of apoptosis, the cells, at a density of 1 × 10^6^ cells/ml, were exposed to 1 μM staurosporine (STS) for 0.5–4 h. All the incubations were done at 37°C.

### Reagents

RPMI 1640 medium and FBS (HyClone Standard) were purchased from Biolot (Russia). STS and ouabain were from Sigma-Aldrich (Germany), and Percoll was purchased from Pharmacia (Sweden). The isotope ^36^Cl^–^ was from “Isotope” (Russia). Salts were of analytical grade and were from Reachem (Russia).

### Experimental Procedures

Details of the experimental methods used were described in our previous study (Yurinskaya et al., 2019). Intracellular K^+^, Na^+^, and Rb^+^ contents were determined by flame emission on a Perkin-Elmer AA 306 spectrophotometer, and the intracellular Cl^–^ was measured using a radiotracer ^36^Cl^–^. Cell water content was estimated by the buoyant density of the cells in continuous Percoll gradient, and it was calculated as *v*_*prot.*_ = (1 - *ρ/ρ*_*dry*_)/[0.72(*ρ* - 1)], where *ρ* is the measured buoyant density of the cells and *ρ*_*dry*_ is the cell dry mass density, which was given as 1.38 g ml^–1^. The share of protein in dry mass was given as 72%. The cell ion and water content were calculated in micromoles per gram of protein.

### Statistical Analysis

Statistical analysis of experimental data was carried out using Student’s *t*-test and is presented in our original publications (Vereninov et al., 2008; Yurinskaya et al., 2011).

### Computation

Computation of the monovalent ion flux balance, membrane potential, and ion electrochemical gradients was performed using the computational program and appropriate executable file BEZ01B as earlier (Vereninov et al., 2014; Yurinskaya et al., 2019). Basic symbols and definitions used are shown in Table 1. The input data (file DataB.txt, in supplement) are the following: extracellular and intracellular concentrations (*na0*, *k0*, *cl0*, and *B0*; *na*, *k*, and *cl*); *kv*; the pump rate coefficient (β); the pump Na/K stoichiometric coefficient (γ); parameter *kb*; channel permeability coefficients (*pna*, *pk*, and *pcl*); and the rate coefficients for the NC, KC, and NKCC cotransporters (*inc*, *ikc*, and *inkcc*). The terms NC, NKCC, and KC, cotransport, or cotransporter, depending on the context, reflect the way these carriers work, but not their genetic identity, which is irrelevant in our study. Therefore, the abbreviations NC and KC are used, but not NCC and KCC. It is known that unidirectional Na^+^–Cl^–^ coupled cotransport with 1:1 stoichiometry may be performed both by a single transport protein, like thiazide-sensitive Na^+^–Cl^–^ cotransporter (Gamba, 2005), and by two functionally coupled exchangers, NHE and Cl^–^/HCO_3_^–^ (Garcia-Soto and Grinstein, 1990; Hoffmann et al., 2009). The genetically identified cotransporters NKCC1 (SLC12A2) and KCC1 (SLC12A4) are expressed in U937 cells^1^,^2^.

**TABLE 1:** Basic symbols and definitions.

**Symbol**	**Definitions and units**
NC, NKCC, KC	Types of cotransporters
[Na]_*i*_, [K]_*i*_, [Cl]_*i*_, *na*, *k*, *cl*	Concentration of ions in cell water, mM
[Na]_*o*_, [K]_*o*_, [Cl]_*o*_, *na0*, *k0*, *cl0*	Concentration of ions in external medium, mM
*B0*	External concentrations of membrane-impermeant non-electrolytes, mM
A	Intracellular content of membrane-impermeant osmolytes, mmol, may be related to g cell protein or cell number
V	Cell water volume, mL, may be related to g cell protein or cell number
V/A	Cell water content per unit of *A*
*z*	Mean valence of membrane-impermeant osmolytes, *A*
OSOR	Ratio of ouabain-sensitive to ouabain-resistant Rb^+^(K^+^) influx
pNa, pK, pCl, *pna*, *pk*, *pcl*	Permeability coefficients, min^–1^
*Beta*, β	Pump rate coefficient, min^–1^
*Gamma*, γ	Na/K pump flux stoichiometry, dimensionless
*kv*	Ratio of “new” to “old” media osmolarity when the external osmolarity is changed
*U*	Membrane potential, MP, mV
PUMP	K^+^ influx or Na^+^ efflux via the pump, μmol⋅min^–1^⋅(ml cell water)^–1^
IChannel, INC, IKC, INKCC	Unidirectional influxes of Na, K, or Cl via channels or cotransport, μmol⋅min^–1^⋅(ml cell water)^–1^
EChannel, ENC, EKC, ENKCC	Unidirectional effluxes of Na, K, or Cl via channels or cotransport, μmol⋅min^–1^⋅(ml cell water)^–1^
*inc*, *ikc*, *inkcc*	Cotransport rate coefficients, ml⋅μmol^–1^⋅min^–1^ for *inc* and *ikc*, and ml^3^⋅μmol^–3^⋅min^–1^ for *inkcc*
*mun*, *muk*, *mucl*	Transmembrane electrochemical potential difference for Na^+^, K^+^, or Cl^–^, mV
*kb*	Parameter of linear decrease of *beta* over time

The rate coefficient of the sodium pump (*beta*) was calculated as the ratio of the Na^+^ pump efflux to the cell Na^+^ content where the Na^+^ pump efflux was estimated by ouabain-sensitive (OS) K^+^(Rb^+^) influx assuming proportions of [Rb]_*o*_ and [K]_*o*_, respectively, and Na/K pump flux stoichiometry of 3:2. *kb* is a parameter of a linear decrease of *beta* over time. Coefficients of ion channel permeability were selected by trial and error, and rate coefficients of cotransporters, *inc*, *ikc*, and *inkcc*, were selected in view of the effects of inhibitors (see text below). Electrochemical gradients for Na^+^, K^+^, or Cl^–^, *mun*, *muk*, or *mucl*, respectively, were calculated by the following equations: *mun* = 26.7⋅ln([Na]_*i*_/[Na]_*o*_) + *U*, *muk* = 26.7⋅ln([K]_*i*_/[K]_*o*_) + *U*, and *mucl* = 26.7⋅ln([Cl]_*i*_/[Cl]_*o*_) - *U* and given in mV. The results of computations appear in the file RESB.txt (its example is given in the supplement). Equivalent exchange fluxes 1:1 are not considered when calculating the ionic homeostasis of the cell, since they do not change the concentration of Na^+^ and Cl^–^ in the cell. Their calculation is considered in our previous study (Vereninov et al., 2016).

## Results

### Normal U937 Cells

#### Measured and Computed Characteristics of the Ion Homeostasis in Cell With NKCC and KC Cotransporters

The measured and computed data obtained for the cell with the NC cotransport only and for the cell with the NKCC and KC cotransports are presented in Table 2. The experimental data are taken from our previous studies (Vereninov et al., 2008; Yurinskaya et al., 2011, and yet unpublished materials). Two variants of normal U937 cells with different measured characteristics, Cells *A* and *B*, are considered to show how computed characteristics depend on the inevitable variability of the real cells. The intracellular concentration of ions; the ratio of ouabain-sensitive to ouabain-resistant influx of Rb^+^(K^+^), OSOR, and their derivatives; the concentration and charge of “impermeable” intracellular anions, A^*z*^, and the pump rate coefficient *beta* belong to the group of “measured” characteristics. The coefficient *beta* is easily and reliably calculated from the measured ouabain-sensitive influx of Rb^+^, the known relationship between the external concentrations of Rb^+^ and K^+^, and the known stoichiometry of the pump, i.e., ratio of the pump K^+^ influx to the pump Na^+^ efflux. Intracellular Na^+^ and Rb^+^ are analyzed in the same sample, and this reduces possible errors. Determination of kinetic parameters characterizing channels and transporters, unidirectional and total fluxes, and membrane potential *U* requires solving differential equations. For this, the original computer program is used (Vereninov et al., 2016; Yurinskaya et al., 2019). The data obtained in this way form a group of “computed” characteristics.

**TABLE 2:** Basic characteristics of ion distribution, measured in two variants of normal resting U937 cells (cells *A* and *B*) and computed for schemes with and without NKCC and KC cotransporters at two chosen sets of NKCC and KC parameters (*X* and *Y*).

**Characteristics**	**Cells *A***			**Cells *B***		
**Measured**						
[K]_*i*_, [Na]_*i*_, [Cl]_*i*_, mM	117, 32, 40			147, 38, 45		
Cell density, g/ml	1.054			1.053		
A^*z*^, mM	121			80		
*z*	−0.90			−1.75		
*beta*	0.029			0.039		
OSOR	5.61			3.89		

**Chosen**	**NC only**	**+ (NKCC and KC)**		**NC only**	**+ (NKCC and KC)**	
		***X***	***Y***		***X***	***Y***

*inc*	3E-5	1E-5	2.8E-5	3E-5	2.5E-5	7E-5
*ikc*	–	2.4E-5	8.8E-5	–	1E-5	8E-5
*inkcc*	–	4E-10	8E-10	–	11.4E-9	8E-9
**Computed**						
*U*, mV	−29.9	−30.8	−34.7	−44.7	−29.4	−45.0
*mucl*	+ 1.5	+2.3	+ 6.3	+19.4	+ 4.1	+19.8
*mun*	−69.3	−70.2	−74.1	−79.5	−64.2	−79.9
*muk*	+ 50.3	+49.4	+ 45.5	+41.6	+ 56.9	+41.3
*pna*	0.0041	0.00355	0.00215	0.00382	0.00535	0.0017
*pk*	0.0115	0.01	0.0058	0.022	0.013	0.0115
*pcl*	0.0125	0.0102	0.005	0.0091	0.028	0.011
**Partial influxes,% of total influx**						
INa Channels	95.1	83.4	53.7	69.3	69.9	28.7
INa NC	4.9	16.2	45.4	30.7	23.0	66.2
INa NKCC	–	0.4	0.9	–	7.1	5.1
IK pump	84.8	84.0	82.8	79.0	79.0	78.0
IK Channels	15.2	13.3	8.0	21.0	9.9	10.9
IK KC	–	2.2	7.9	–	0.5	4.2
IK NKCC	–	0.6	1.2	–	10.5	6.9

*The concentration of ions, impermeant intracellular anions A, their charge z, pump rate coefficient beta, and OSOR—ratio of ouabain-sensitive to ouabain-resistant components of K^+^(Rb^+^) influx—are determined as described in section “Materials and Methods.” Symbols, definitions, and units are given in Table 1. Partial fluxes are given in% to the sum of fluxes, excluding a 1:1 exchange flux. Cell variants A and B differ due to the use of different subline U937 cells studied in different years. The experimental data are taken from our previous study (Vereninov et al., 2008; Yurinskaya et al., 2011, and yet unpublished materials).*

The “computed” characteristics depend not only on the experimental data used but also on the chosen list of ion pathways and on the relationship between the parameters characterizing the properties of the pathways. Shown are two different sets of parameters, *X* and *Y*, for two different cell variants, *A* and *B*. Both sets of *X* and *Y* give cells with almost the same characteristics, like those measured in real cells. Thus, it can be assumed that real cells can also achieve the same monovalent ion homeostasis in different ways. The number of solutions to the flux balance equations in a cell with only NC cotransport was discussed earlier (Yurinskaya et al., 2019). The introduction of the NKCC and KC cotransporters into consideration increases the number of possible solutions to the system of equations describing cell ion homeostasis. Parameters *X* and *Y* are chosen as examples of possible differences that result in unidirectional NKCC and NC fluxes in the range possible for real U937 cells, as follows from the data on the effects of NKCC and KC inhibitors. We are currently unable to determine which of the *X* or *Y* options is more appropriate for real cells. However, the computation can show what additional measured characteristics can help to select the best option. For example, variants *B*–*X* and *B*–*Y* differ significantly in the resting membrane potential, in the K^+^(Rb^+^) influx sensitive to inhibitors, in the electrochemical Cl^–^ gradient, and, consequently, in the effect of changes in the Cl^–^ channel permeability on the entire ionic homeostasis. Variants *A–X* and *A–Y* differ significantly in K^+^(Rb^+^) influx sensitive to the specific NKCC and KC inhibitors, bumetanide, and DIOA, respectively. The difficulty here lies in the low values of the partial fluxes NKCC and KC in U937 cells (see below). The parameters of the main ion pathways computed at different variants of the NKCC and KC rate coefficients are different. However, the range of their variation can be estimated by computing the desired options.

#### Ion Fluxes

**FIGURE 1:**
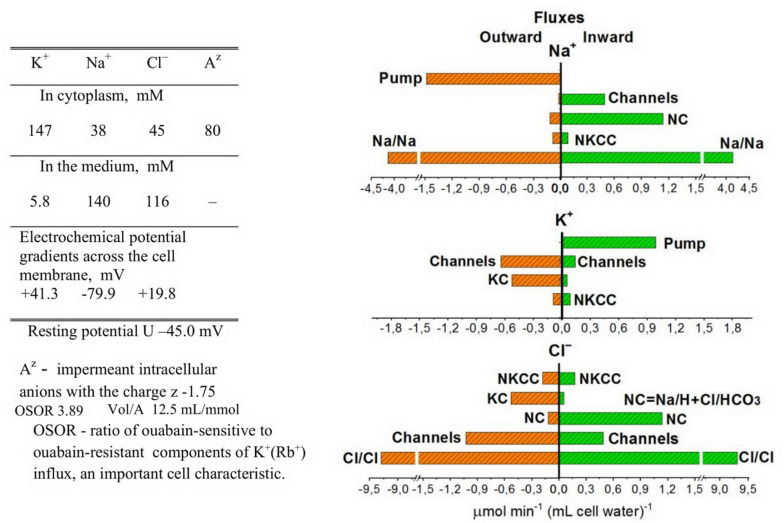
Ion distribution and unidirectional K^+^, Na^+^, and Cl^–^ fluxes in normal U937 cells under the balanced state. Cells *B*–*Y* from Table 2 are given as an example. Other details are on the plot.

Figure 1 shows the relationship between all quantitatively significant components of the unidirectional fluxes of Na^+^, K^+^, and Cl^–^ in U937 cells (*B*–*Y*) calculated on the assumption that seven types of ionic pathways exist in their plasma membrane in a balanced cell state: the Na/K ATPase pump; the Na^+^, K^+^, and Cl^–^ electroconductive channels; the NC, NKCC, and KC cotransporters; and the coupled ion exchange with a 1:1 stoichiometry, which is especially important in consideration of Na^+^ and Cl^–^ unidirectional fluxes. The equivalent exchange Na/Na and Cl/Cl constitutes the dominant part of the entire unidirectional flux of these ions through the cell membrane. These exchange fluxes are not associated with changes in intracellular ion concentrations and differences in electrical potentials in the cell membrane or with its conductivity and cannot be measured by electrophysiological methods. However, they are extremely significant when the transport of ions across the membrane is studied by measuring fluxes using radioisotopes. These measurements show that the fluxes underlying the 1:1 exchange of ions across the membrane can be significantly higher in real cells than those considered in a simple pump-leak model. The presented data on the overall exchange fluxes of Na^+^ and Cl^–^ in the normal U937 cells have been obtained using radiotracers ^22^Na^+^ and ^36^Cl^–^ or low concentrations of Li^+^ as an analog of Na^+^ for most of the Na^+^ pathways except the pump (Vereninov et al., 2007, 2016; Yurinskaya et al., 2011). The dominance of the 1:1 exchange flux in the entire Cl^–^ flux across the plasma membrane is known for other cells (Hoffmann et al., 1979; Hoffmann, 1982, 2001). Fluxes related to the Na/Na and Cl/Cl equivalent exchange are not considered further in the calculation of the flux balance.

Computation shows that the effect of the NKCC cotransport on the asymmetry in the balanced distribution of K^+^, Na^+^, and Cl^–^ in U937 cells is small, because the influx and efflux of ions mediated by NKCC differ insignificantly and the net NKCC flux is small in comparison with other net fluxes (Table 3). Importantly, the unidirectional NKCC influx accounts for 0.4 and 0.9% of the total Na^+^ influx in cells *A* but 7.1 and 5.1% in cells *B* in variants *X* and *Y*, respectively (Table 2). Detection of such a small bumetanide-inhibitable NKCC influx in the presence of a large background Na/Na equivalent exchange is impossible. The Na^+^ net flux via NKCC is small as the driving force for NKCC is small. The driving force for NKCC cotransport is *mun* + *muk* + 2*mucl* = −16.2 ÷−16 mV in cells *A* and + 0.9 ÷ + 1 mV in cells *B* (Table 2).

**TABLE 3:** Net and unidirectional K^+^, Na^+^, and Cl^–^ fluxes in normal resting U937 cells calculated for two variants of cells and for two different sets of cotransport parameters, *X* and *Y*.

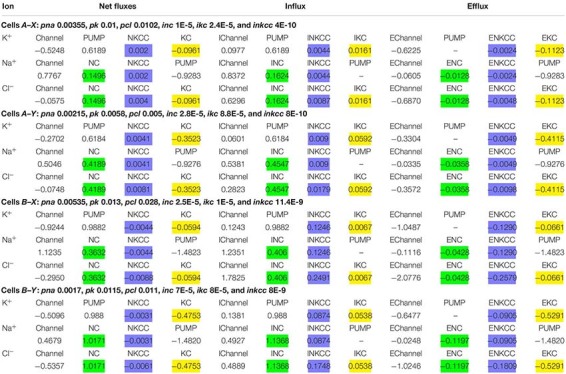

*Basic ionic characteristics of cells A and B and parameters X and Y are given in Table 2. Parameters in computation: Cells A, na 32, k 117, cl 40, and beta 0.029; Cells B, na 38, k 147, cl 45, and beta 0.039. The following parameters are the same for both variants: na0 140, k0 5.8, cl0 116, B0 48.2, gamma 1.5, kv 1.0, hp 240, and kb = 0. Specific sets of channel permeability coefficients and cotransporter parameters are shown in the table. Fluxes are given in μmol⋅min^–1^⋅(ml cell water)^–1^. Calculation performed as described in section “Materials and Methods.” Cells under the balanced state and Cl^–^ and Na^+^ fluxes via exchangers 1:1 are omitted.*

The balanced unequilibrium distribution of Na^+^ (*mun* is −64 ÷ -80 mV depending on variants) is determined mostly by the relationship between the Na^+^ pump flux uphill and Na^+^ flux downhill through not only the channels but also via the NC pathway. This means that the Na^+^ flux through the NC route significantly “loads” the pump in the studied U937 cells. Computation shows that NC cotransport is a strong regulator of intracellular Na^+^ concentration with all the ensuing consequences.

A small share of KC influx in the entire K^+^ influx in the considered cells (0.5–7.9% depending on the options) (Table 2) makes it difficult to study KC fluxes with inhibitors. At the same time, the net K^+^ flux via KC can be comparable with the net K^+^ flux through the channels (see examples *A*–*Y* and *B*–*Y*, Table 3). Therefore, the effect of KC cotransporter on the entire cell ion homeostasis can be significant.

Computation makes it possible to quantify the effect of the NC, NKCC, and KC cotransporters on generation of the Cl^–^ electrochemical gradient across the cell membrane. The NC cotransport is the most important here, at least in U937 cells. KC and NC cotransports are antagonists as the net Cl^–^ flux due to the KC cotransport is directed out of the cell whereas that due to the NC cotransport, in contrast, is directed into the cell (Table 3). The movement of Cl^–^ into the cell due to NC or from the cell due to KC leads to a non-equilibrium distribution of Cl^–^ across the membrane in a balanced state and changes the apparent content of “impermeant” anions in the cell. According to the theory of the double Donnan system, the amount of “impermeant” anions in a cell is a basic factor creating the asymmetry in distribution of ions across the cell membrane and the electrical potential difference at the membrane. Thus, NC and KC cotransporters turn out to be important regulators of the ionic, electrical, and water balance of the cell as a whole due to their influence on the unequilibrium distribution of Cl^–^. The Cl^–^ unequilibrium distribution also makes the Cl^–^ channel permeability an important regulator of the entire ionic homeostasis of the cell. The interaction of the NC, NKCC, and KC cotransporters and Cl^–^ channels is well tested using our computational program.

### Apoptotic Changes in the Net and Unidirectional Fluxes of Na^+^, K^+^, and Cl^–^, Underlying the Change in Ionic and Water Balance in U937 Cells Treated With STS

**FIGURE 2:**
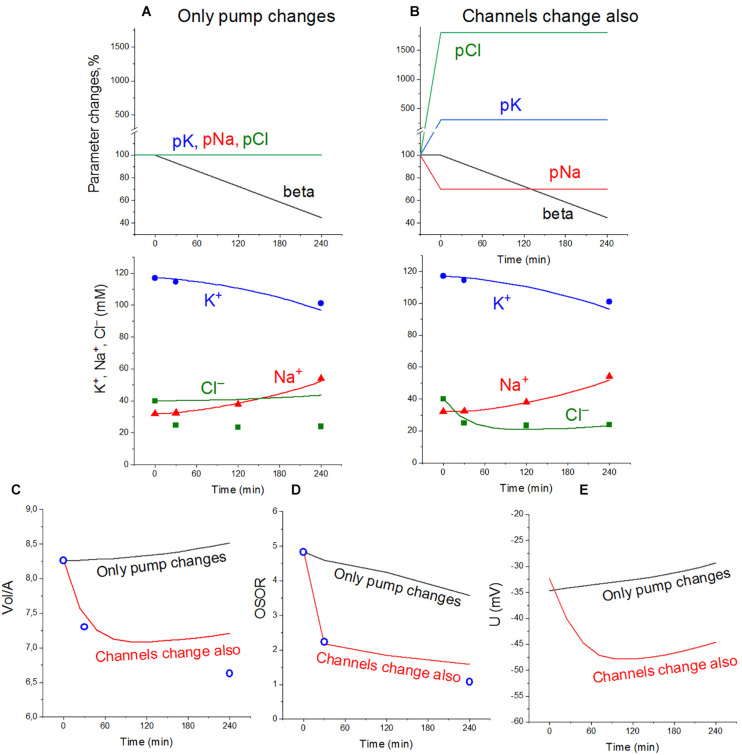
Apoptotic changes in measured and calculated intracellular concentrations of Na^+^, K^+^, and Cl^–^ **(A,B)**, cell water Vol/A **(C)**, OSOR **(D)**, and calculated membrane potential *U* **(E)** in U937 cells. Cells *A*–*Y* from Table 2 are given as an example of apoptotic cells treated with STS. Experimental data are shown by symbols; calculated, by lines. **(A)** Only a linear decrease of the pump rate coefficient *beta* from an initial 0.029–0.013 at 4 h (coefficient *kb* = 0.000068 *t* > 0). **(B)** Additional changes in channel permeability shown on the top graphs: *pna* 0.00215*_*t*_*_= 0_→0.0015*_*t*_*_> 0_; *pcl* 0.005*_*t*_*_= 0_→0.09*_*t*_*_> 0_; *pk* 0.0058*_*t*_*_= 0_→0.0174*_*t*_*_> 0_; *inc*, *ikc*, and *inkcc* rate coefficients remain constant throughout. Initial parameters: *na0* 140; *k0* 5.8; *cl0* 116; *B0* 48.2; *kv* 1.0; *na* 32; *k* 117; *cl* 40; *beta* 0.029; *gamma* 1.50; *pna* 0.00215; *pk* 0.0058; *pcl* 0.005; *inc* 2.8E-5; *ikc* 8.8E-5; and *inkcc* 8E-10.

The dynamics of apoptotic changes in the measured characteristics of ionic balance in U937 cells treated with STS, and changes in the rate coefficients for the main transporters and channels, calculated for the cell with the NKCC and KC cotransporters are shown in Figure 2. The measured linear decrease in the rate coefficient of the Na/K pump remains an important factor in the apoptotic alteration of cell ionic balance in the cell with NKCC and KC like in the cell with NC only. It should be noted that the pump K^+^ and Na^+^ fluxes in apoptotic cells decrease with time more slowly than the pump rate coefficient beta due to an increase in intracellular Na^+^ during apoptosis (Table 4). This is a good example that the pump coefficient *beta* is a more adequate characteristic of the intrinsic properties of the pump. The data in Figure 2A show that a decrease in pump activity gives good agreement between calculated and experimental results for K^+^ and Na^+^, but not for Cl^–^, cell water, and OSOR (Figures 2C,D). Changes in the permeability coefficients of ion channels are required.

**TABLE 4:** Dynamics of the net and unidirectional K^+^, Na^+^, and Cl^–^ fluxes during apoptosis of U937 cells.

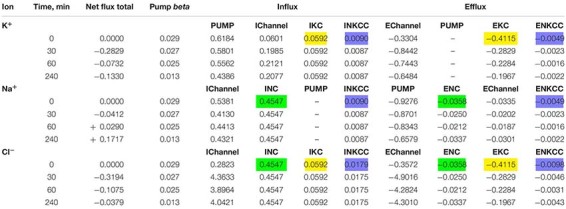

*Cells A and set of parameters Y are shown as an example. STS is introduced in the media at t = 0. Fluxes are given in μmol⋅min^–1^⋅(ml cell water)^–1^. Calculation is performed as described in section “Materials and Methods.” Basic characteristics of cells and parameters are given in Table 2. Parameters change at t > 0: pna 0.00215 to 0.0015; pk 0.0058 to 0.0174; pcl 0.005 to 0.09; kb 0_*t=0*_ to 0.000068_*t*__>__0_; inc, ikc, and inkcc remain unchanged; see Figure 2. Cl^–^ and Na^+^ fluxes through 1:1 exchangers are not included in the calculation.*

Changes in pCl, pK, and pNa, which give good agreement between the calculated and observed dynamics of the ion and water content during apoptosis, turn out to be practically the same (qualitatively) as in the cell without NKCC and KC in *A*–*X* cells, but with more significant changes in pCl in *A*–*Y* cells (Table 5). The experimental data obtained for the studied U937 cells do not allow one to accurately determine the changes in parameter values but are sufficient to determine the extent of possible parameter changes. Our conclusion is that apoptotic changes in unidirectional and net fluxes of Na^+^, K^+^, and Cl^–^ in the studied U937 cells are caused by a gradual decrease in the pumping coefficient by about a factor of 2 in 4 h, steep increase in the integral permeability of the Cl^–^ channel by 6–18 times (depending on the selected cell), increase in the permeability of the integral K^+^ channel by about 3 times, and decrease in the permeability of the integral Na^+^ channel by 30%. The introduction of the KC and NKCC cotransporters into the calculations did not change the general mechanism of apoptotic changes in ionic homeostasis.

**TABLE 5:** Changes in the permeability coefficients of Na^+^, K^+^, and Cl^–^ channels obtained for apoptotic U937 cells by computation at a different set of parameters of NC, KC, and NKCC cotransporters (cells *A* in Table 2).

**Cotransporters**		**Channel permeability**	**Resting**	**In apoptosis**	**Apoptotic to resting**
NC only		pCl	0.0125	0.068	5.4
		pNa	0.0041	0.003	0.73
		pK	0.0115	0.030	2.6
NC, KC, and NKCC	*X*	pCl	0.0102	0.065	6.4
		pNa	0.00355	0.0025	0.70
		pK	0.010	0.033	3.3
	*Y*	pCl	0.005	0.09	18
		pNa	0.00215	0.0015	0.70
		pK	0.0058	0.0174	3.0

## Discussion

The importance of cotransporters in maintaining cellular ion homeostasis and active Cl^–^ transport against an electrochemical gradient is generally known (Russell, 2000; Hoffmann et al., 2009; Lang and Hoffmann, 2012, 2013; Jentsch, 2016; Delpire and Gagnon, 2018; Dmitriev et al., 2019). However, only a few studies have considered the cotransporters in the quantitative description of the entire balance of ion fluxes across the cell membrane. Lew was the first to find that the balance in human reticulocytes with a non-equilibrium balanced distribution of Cl^–^ cannot be explained without NC (Lew et al., 1991). NC and KC cotransporters were included in cellular ionic homeostasis model by Hernández and Cristina (1998). The NKCC cotransport was considered when modeling the ionic balance in cardiomyocytes (Terashima et al., 2006). The balance of Cl^–^ fluxes through cotransporters and channels was not considered in the early fundamental studies of cotransporters in erythrocytes because the distribution of Cl^–^ is determined in these cells by a powerful Cl^–^/HCO_3_^–^ exchanger, but not by Cl^–^ channels.

Later, the interplay between various cation-coupled Cl^–^ cotransporters (SLC12 gene family) and Cl^–^ channels has been intensively studied in relation to signal transduction in neurons (Kaila et al., 2014; Doyon et al., 2016; Currin et al., 2020; Wilke et al., 2020), in astroglia physiology (Wilson and Mongin, 2018) and in phagocytosis (Wang, 2016; Perry et al., 2019). Unfortunately, in all these cases, it was extremely difficult to obtain a complete set of accurate and reliable experimental data necessary for quantitative description of the entire flux balance across the cell membrane. Computer calculations show that small variations in the input data may produce substantially different results.

The use of ion-sensitive dyes and calcein to determine the cell water, Cl^–^, and Na^+^ allows us to measure mainly the relative changes in their values, but absolute value measurements may not be reliable enough due to calibration difficulties. Recently, we have found that measurements of Na^+^ concentration in U937 cells by the direct flame emission method and by the ANG-2 dye method give substantially different results (Yurinskaya et al., 2020b).

It is important that U937 cells cultured in suspension allow the determination of the cell water content by measuring the buoyant density without known problems in estimation of extracellular water in the sample. These cells also allow determining cell ions both by direct flame emission and by ion-sensitive dyes using flow cytometry. It is also important that cells cultured in suspension remain intact during transfer to a flow cytometer or to a density gradient. The detachment of cells from the substrate, enzymatically or mechanically, when working with adherent cell cultures, always affects their ionic composition. We use computer calculations and cell experimental data to analyze the role of the NKCC and KC cotransporters in the ionic balance of cells at rest and in early apoptosis. Our program allows one to quickly test various sets of parameters characterizing the kinetics of ion transport through multiple pathways of the cell membrane. By combining measured and computed data, we were able to compare pathway behavior in normal and apoptotic cells.

One of the most important and difficult problems in this area is the determination of the real values of the parameters based on real experimental data, rather than searching additional parameters characterizing all variety of channels or transporters. To quantitatively analyze the relationships between fluxes through multiple ionic pathways in the cell membrane, it is sufficient to use the driving forces for each type of pathway that are known and linear coefficients for each pathway and each type of ion. The number of even simplified parameters turns out to be rather large. In our case, it is impossible to predict theoretically how many sets of parameters will give the same result and how to obtain a unique set of parameters that provide agreement between experimental and calculated data (Yurinskaya et al., 2019). However, a unique set of parameters can be obtained if auxiliary information from the experiment is used in addition to mathematical solutions. In the present study, the ratio of ouabain-sensitive to ouabain-resistant components of rubidium influx (OSOR) helped to select the right set of parameters. OSOR is easily and reliably obtained from experimental data. Its value is included in the output table calculated by our computing program. The use of bumetanide and DIOA, specific blockers of NKCC and KC fluxes, gave an upper limit for Rb^+^(K^+^) influxes mediated by these transporters in our study. Calculations predict different membrane potential for a different set of NKCC and KC parameters. Hence, the measurement of membrane potential can determine which of the *X* or *Y* parameter sets in Table 2 is correct for the cells in question. We suggest that reliable membrane potential data can be obtained using voltage-sensitive dyes such as DiBaC4 rather than microelectrodes. An interesting comparison of the two methods has been made recently (Bonzanni et al., 2020). Measuring multiple cell characteristics can solve the problem of multiple parameters.

U937 cells are an established model in the experimental study of apoptosis. The calculation of apoptotic changes in cell ionic homeostasis in our previous study concerned the interaction between pump, channels, and NC cotransport without NKCC and KC. The effects of the cotransporters NKCC and KC on changes in the flux balance during apoptosis remained unexplored. Calculation of the net and unidirectional Na^+^, K^+^, and Cl^–^ fluxes via KC and NKCC pathways in the studied cells showed that the difference in the effects of the specific inhibitors of KC and NKCC on the uptake Rb^+^(K^+^) as in conventional methods of testing KC and NKCC cotransport does not reflect their impact upon the entire ionic homeostasis. The effect of the net KC flux on the entire ionic balance is higher than the net NKCC flux because the driving force in the KC pathway is higher than in the NKCC pathway. Whereas NC plays a critical role in generating *mucl*, the driving force for the Cl^–^ net flux through channels with all its consequences, the NKCC cotransport is practically ineffective in creating *mucl*, while KC can only reduce it.

An increase in the permeability of Cl^–^ channels at the early stage of apoptosis, found by calculations, is in good agreement with numerous electrophysiological data indicating an increase in the integral conductance of chloride channels VRAC during apoptosis (Hoffmann et al., 2015; Kondratskyi et al., 2015; Jentsch, 2016; Pedersen et al., 2016; Wanitchakool et al., 2016). We studied the expression of these channels in U937 cells under our experimental conditions using an antibody against the outer loop of the LRRC8A VRAC subunit (Yurinskaya et al., 2020a). It was found that the number of channels expressed in the membrane does not change to the extent comparable with the changes in the integral permeability of Cl^–^ channels. This indicates a change in the internal properties of the channels, but not their number in the membrane.

Calculation shows that the net fluxes out of cells of all three ions, K^+^, Na^+^, and Cl^–^, arise at the early stage of apoptosis (Table 4). The K^+^ net flux out of cells is the one of the most significant causes of the known apoptotic decrease of K^+^ content in cells and their concomitant apoptotic shrinkage (Yurinskaya et al., 2005). The significant K^+^ net flux out of cells arises at the early stage of apoptosis mostly due to an increase of the channel K^+^ efflux caused by an increase in channel permeability and also due to a decrease of the pump K^+^ influx. It should be noted that the K^+^ and Cl^–^ channel fluxes increase in both directions, but the increase in efflux exceeds the increase in influxes. The net Cl^–^ flux out of cells underlies the initial drop in Cl^–^ content and concentration observed in apoptotic cells. It is caused not only by an increase of pCl from 0.005 to 0.09 but also by a shift in the membrane potential toward hyperpolarization due to an increase in pK. The shift in the membrane potential causes an increase in Cl^–^ electrochemical potential difference. The initially outward net Na^+^ flux reverses later to the inward net flux and Na^+^ content, and concentration in apoptotic cells begins to increase. This is because the apoptotic decrease in the pump efflux outweighs the decrease in channel Na^+^ influx (Table 4). It has been shown earlier that pNa decrease and pK increase alone without a pCl increase could also be sufficient to get agreement between real and calculated chloride concentrations for the first 30 min. However, this variant should be rejected as the OSOR value becomes unrealistically low (Yurinskaya et al., 2019).

Non-zero net fluxes of all three K^+^, Na^+^, and Cl^–^ ions in U937 cells during STS-induced apoptosis indicate that a balanced ion distribution is not achieved within the first 4 h. In our earlier study (Yurinskaya et al., 2011), it was hypothesized that a balanced state takes place in apoptotic U937 cells under the discussed conditions. At that time, data on the distribution of Cl^–^ were scarce, and the computational tool was not yet sufficiently developed. Based on that hypothetical assumption it was concluded that “A decrease in the channel permeability of the plasma membrane for Na^+^ is proved to be crucial for preventing cell swelling due to the decrease in the Na^+^/K^+^ pump activity in cells undergoing apoptosis whereas opening of the K^+^ and Cl^–^ channels is not required”. This conclusion should be considered at present as incorrect.

It would be very interesting to learn about the role of monovalent ions not only at the early 4 h stage of STS-induced apoptosis in U937 cells, despite the fact that this model is popular and that the developed approach to studying apoptosis in other cells may have a more general significance. The role of monovalent ions in the subsequent development of apoptosis is no less interesting, since understanding apoptosis in its entirety is necessary for solving many practical problems, for example, searching for targets of anticancer drugs. Unfortunately, there are still no necessary data on changes in the ion content, water balance, and sodium pump fluxes at the late stages of apoptosis. A quantitative description of ionic processes during late apoptosis is a matter of the future.

## Conclusion

A developed approach enables us to obtain a complete list of the inward and outward Na^+^, K^+^, and Cl^–^ fluxes via all major pathways across the plasma membrane including NKCC and KC cotransporters in U937 cells at rest and during the first 4 h of apoptosis induced by staurosporine.

The problem of the inevitable multiplicity of solutions to the flux equations arising with an increase in the number of paths for ions can be solved in real cases if we take into account the ratio of the ouabain-sensitive and ouabain-resistant parts of the influx K^+^(Rb^+^) and use additional experimental data on the effects of specific inhibitors or some other data.

The dynamics of changes in plasma membrane channels and transporters, which underlie apoptotic changes in the content of ions and water in cells, calculated earlier without taking into account the KC and NKCC cotransporters, differs from that calculated for cells with the KC and NKCC cotransporters only in details.

The developed approach to determining unidirectional fluxes can be useful for studying the functional expression of ion channels and transporters in other cells.

## Data Availability Statement

The original contributions presented in the study are included in the article/Supplementary Material, further inquiries can be directed to the corresponding author.

## Author Contributions

AV wrote the manuscript with input from all authors. All authors contributed to the design of the experiments, performed the experiments, analyzed the data, and approved the final version of the manuscript and agreed to be accountable for all aspects of the work. All persons designated as authors qualify for authorship, and all those who qualify for authorship are listed.

## Conflict of Interest

The authors declare that the research was conducted in the absence of any commercial or financial relationships that could be construed as a potential conflict of interest.

## Footnotes

1 https://www.proteinatlas.org/ENSG00000064651-SLC12A2/blood

2 https://www.proteinatlas.org/ENSG00000124067-SLC12A4/blood
