# Supplementary material for: Balance of Na+, K+, and Cl– Unidirectional Fluxes in Normal and Apoptotic U937 Cells Computed With All Main Types of Cotransporters
Source: Front Cell Dev Biol. 2020 Nov 6;8:591872. doi: 10.3389/fcell.2020.591872 (PMC7677585; doi:10.3389/fcell.2020.591872)
Supplement: Supplementary file 1 [file Data_Sheet_1.ZIP › How to use the executable file to programme BEZ01BM.docx]

How to use the executable file to the programme BEZ01BM.

1. The executable file BEZ01BM (BEZ01BM.txt when e-mailed) is best used on a 32-bit

computer with Windows OS. The user should in this case:

a. Locate files DATAB.txt and BEZ01BM.txt in the same folder (DATAB control txt with deleted “control” is used for checking the procedure).

b. Change the extension of BEZ01BM from “TXT” to “exe” (do not try to open BEZ01BM.txt. It is unreadable!).

c. Run the executable file and wait until the process is completed and the file RESB.txt appears.

d. Rename and save the obtained file RESB.txt because in a new running cycle it will be lost.

The resulting output file should be the same as in the file RESB control.txt if the DATAB control.txt (with deleted “control”) was used. If it is OK, the process with selected parameters in DATAB.txt that is interesting to the user can be repeated. Instead of zero values for concentrations, small non-zero values should be used in DATA.txt (e.g., 0.001 mM). Do not forget that after varying DATAB, the command “save” should be done. The word “control” in the control file name should be deleted during checking.

2. To run the executable file on a 64-bit machine, the following additional steps should be taken:

e. Download the School Pak package via Internet and run Norton Commander (NCD).

f. Set in NCD the same folder as the folder in Windows where the DATA.txt and executable file BEZ01BM are located.

g. Correct the file DATA.txt if necessary in the Windows folder.

i. Run executable file in the NCD folder and read RESB.txt in the analogous Windows folder.
